# Supplementary material for: Localized statistics decoding for quantum low-density parity-check codes
Source: Nat Commun. 2025 Sep 2;16:8214. doi: 10.1038/s41467-025-63214-7 (PMC12405505; doi:10.1038/s41467-025-63214-7)
Supplement: Supplementary file 1 — Supplementary Information [file 41467_2025_63214_MOESM1_ESM.pdf]

Supplementary Information for “Localized statistics decoding for quantum low-density parity-check codes"

Timo Hillmann,[1] Lucas Berent,[2] Armanda O. Quintavalle,[3]
Jens Eisert,[3,4] Robert Wille,[2,5] and Joschka Roffe[3,6]

[1] *Chalmers University of Technology, Gothenburg, Sweden*
[2] *Technical University of Munich, Munich, Germany*
[3] *Freie Universität Berlin, Berlin, Germany*
[4] *Helmholtz-Zentrum Berlin für Materialien und Energie, Berlin, Germany*
[5] *Software Competence Center Hagenberg, Hagenberg, Austria*
[6] *University of Edinburgh, Edinburgh, United Kingdom*

Supplementary Note 1 – Clusters on decoding graphs

The primary motivation behind the LSD algorithm is to divide the decoding problem into smaller, more manageable, sub-problems. This section investigates the conditions under which such a division is feasible. We study the structure of clusters that form in decoding graphs of QLDPC codes under an independent and identically distributed noise model, and we relate this structure to the runtime of the parallel LSD algorithm.

As discussed in the main text, a check matrix H – whether derived from a stabilizer code or its syndrome extraction circuit – can be interpreted as the incidence matrix of a bipartite graph, $\mathcal{G}(H) = (V_D \cup V_F, E)$, which we refer to as the Tanner graph of H. Although the Tanner graph encapsulates all the information necessary to encode the decoding problem, it is beneficial to consider the projection of the Tanner graph onto the fault nodes V_F when investigating the statistical distribution of errors under a determined noise model. We call this projected graph, with vertices V_F, the *fault graph*, and denote it as $\mathcal{F}(H)$. Two vertices $v, v' \in V_F$ are connected by an edge in $\mathcal{F}(H)$ if and only if v and v' have a common neighbor in $\mathcal{G}(H)$, i.e., if and only if there exists $d \in V_D$ such that (d, v) and (d, v') are edges in $\mathcal{G}(H)$.

To model an error distribution on the fault graph $\mathcal{F}(H)$, we assume that each fault node is occupied randomly with probability p and empty with probability $1-p$. Hence, for n faults, we expect np faults to be occupied, and $n(1-p)$ to be empty. This setup defines a site percolation problem, which involves studying the distribution of groups of neighboring occupied faults, called *clusters*. We call any instance of the site percolation problem an *error* defined on the fault graph $\mathcal{F}(H)$, and we expect clusters to have varying sizes and shapes depending on both the fault graph connectivity and the flip probability p.

Crucially, for low enough error probability p, random errors are likely composed of small disjoint clusters on the fault graphs of bounded LDPC matrices H, and hence yield disjoint and independent decoding problems that can be solved locally [1, 2]. This argument ultimately serves as the proof that (r, c)-bounded LDPC codes, with distance scaling as $\sim n^\alpha$ for $\alpha > 0$, have a threshold [1, 3, 4]. In this section, we use similar statistical tools to estimate the expected performance of the LSD decoding algorithm.

Knowledge of the cluster structure of an error would enable an exact and optimal factorization of the decoding problem into smaller sub-problems. LSD initializes one cluster for each activated detector node on the Tanner graph and uses prior knowledge of the noise model and the fault graph structure to grow each cluster until it is valid, i.e., until it defines a solvable decoding problem. Provided that fully grown clusters cover the original error clusters, the computational cost of a parallel implementation of LSD is bounded by the computational cost of solving the decoding problem over the biggest cluster found.

In the best scenario possible, the guided cluster growth is perfect, meaning that the clusters grown by LSD match exactly the actual error clusters. In this scenario, the running time of serial LSD is proportional to

1. the number ν of clusters of the error and

2. the size κ of the largest cluster of the error.

More broadly, the expected performance depends on the average size of the clusters of the error.

In general, if we assume sufficient parallel resources, the LSD algorithm can use ν cores and run in $O(\kappa^3)$ time in the worst case, where κ represents the size of the largest cluster in a given instance. However, from a practical perspective, the largest cluster size for each sampled error (shot) is not the sole relevant figure of merit. As our statistical analysis suggests and our numerical investigation confirms, most errors exhibit minimal variance in their cluster size distributions, meaning that the clusters have the same size up to small fluctuations. Consequently, a useful practical proxy for the expected parallel runtime of LSD is the average cluster size per shot, κ_α, rather than the worst-case cluster size.

Following this reasoning, we collect statistical data on these three quantities ν, κ and κ_α, for a given noise model of interest. We study the expectation value of ν, κ and κ_α for:

(i) errors sampled according to the noise distribution on the fault graph;

(ii) final clusters found by BP+LSD on termination of the algorithm that is, when all clusters are valid.

The optimal LSD implementation is such that the statistics found at point (ii) match the one found at point (i).

In Figure 3 of the main text, we report the statistics for the fault graph obtained from the circuit-level noise simulation of the $[\![144, 12, 12]\!]$ code [5]. As we can see, for noise values below the threshold, there is no statistical difference between the two different cluster distributions, indicating that the clusters found by BP+LSD are of minimal size and hence (close to) optimal.

A. Analytic cluster bounds

In the remainder of this section, we discuss some known analytical site percolation results on the cluster distribution on regular graphs. Instead of investigating average clusters properties via statistical tools as proposed above, (see, e.g., Figure 3 of the main text), we present some upper bounds, which mainly make use of approximation of regular graphs as regular trees [6, 7].

If H is an LDPC matrix with constant row and column weight r and c, respectively, the associated fault graph $\mathcal{F}(\mathcal{H})$ is a regular graph of degree $\theta = c(r-1)$, with $\theta \cdot n/2$ edges. Let $n_s^p(v)$ be the number of clusters of size s containing the vertex v. In general, this takes the form

$$p^s(1-p)^{|\text{perimeter}|}, \tag{1}$$

where the perimeter of a cluster is the set of non-occupied nodes adjacent to at least one node in the cluster. The probability that the node v belongs to a particular cluster of size s is $n_s^p(v)s$, as v can be any of the s nodes in the clusters. On our percolation problem, by assumption, the probability that a given node is occupied is p. Moreover, if a node is occupied, it belongs to a cluster of size $\tilde{s}$ for some $\tilde{s} = 1, \ldots, n$, where n is the number of nodes in the graph. Hence, for a given node in the graph, the probability p of being occupied must equate the sum of the probabilities of belonging to a cluster of size $\tilde{s} = 1, \ldots, n$. Hence, for an arbitrary node in the graph, it holds that

$$\sum_s n_s^p(v)s = p. \tag{2}$$

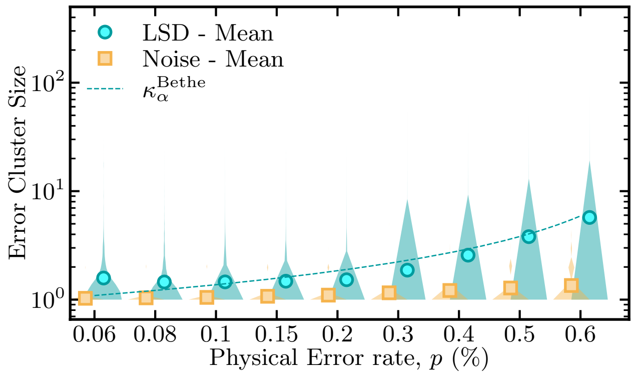

Supplementary Figure 1. Distribution of the cluster size statistics of the $[\![144, 12, 12]\!]$ bivariate bicycle code of Ref. [5] under circuit-level noise with strength p. Markers show the mean of the distribution while shapes are violin plots of the distribution obtained from 10^5 samples. Yellow distributions show statistics for the optimal factorization while the blue distributions show statistics for the factorization returned by BP+LSD. The dashed line represents the expected average cluster size for the Bethe lattice, κ_α, for $\theta = 139$. Markers and distributions are slightly offset from the actual error rate to increase readability.

That is, the probability $n_s^p(v)$ that a node belongs to an s-cluster times the number of nodes/choices for that cluster. A quantity of interest for decoding is an estimate of the size of the cluster a random node belongs to. In other words, the probability w_s that a node is occupied and belongs to an s-cluster. Explicitly, using Equation (2), we find

$$w_s = \frac{n_s^p(v)s}{p} = \frac{n_s^p(v)s}{\sum_{\tilde{s}} n_{\tilde{s}}^p(v)\tilde{s}}, \tag{3}$$

and therefore the average cluster size is given by

$$\begin{aligned} \kappa_\alpha &= \sum_s w_s s \\ &= \frac{1}{p}\sum_s n_s^p(v)s^2. \end{aligned} \tag{4}$$

For a general graph of bounded degree θ, these quantities are difficult to estimate. The distribution of cluster sizes takes asymptotically the form $s^{-\tau}e^{-s/s_\xi}$ for large sizes s [7], with some characteristic cluster size s_ξ and for a suitable $\tau > 0$, but again, for our purposes, they are not easy to assess.

Nonetheless, we can find some upper bounds by looking at the same percolation problems on the Bethe lattice. The Bethe lattice is an infinite tree where all vertices have the same degree. For instance, on each θ-bounded graph, the size of the perimeter of a s-cluster is upper-bounded by the size of the perimeter of an s-cluster on the Bethe lattice of degree θ, that is the same for all s-clusters and it is maximal across the perimeters of s-clusters for θ-bounded graphs, taking the value $(\theta - 2)s + 2$.

For the Bethe lattice, we can use self-similarity to compute the average cluster size $\kappa_\alpha^{\text{Bethe}}$. If v is occupied, we can think of the cluster it belongs to as made up of the node v itself plus the sub-clusters, i.e., subsets of connected vertices in that cluster, rooted in its occupied neighbors. Each occupied neighbor contributes to the cluster rooted in v with itself (add 1) and the sub-clusters not rooted in v itself, which at most $\theta - 1$;

by self-similarity, these sub-clusters have same average size as the cluster rooted in v, and contribute to the count adding at most $(\theta-1)\kappa_\alpha^{\text{Bethe}}$ vertices. Thus, if the vertex v is occupied, it belongs to a cluster of size $1+(\theta-1)\kappa_\alpha^{\text{Bethe}}$. As each neighbor of the vertex v is empty with probability $(1-p)$, and occupied with probability p, we find

$$\kappa_\alpha^{\text{Bethe}} = p\left(1+(\theta-1)\kappa_\alpha^{\text{Bethe}}\right), \tag{5}$$

and via substitution:

$$\kappa_\alpha^{\text{Bethe}} = \frac{p}{1-p(\theta-1)}. \tag{6}$$

In conclusion, provided that the decoding problem of our interest is defined on a parity check matrix of bounded column and row weight, we can upper bound the expected average cluster size with the expected average cluster size $\kappa_\alpha^{\text{Bethe}}$ on the Bethe lattice [6, 7].

As an example of this upper bound via the Bethe lattice, in Supplementary Figure 1, we plot cluster size distributions against the physical error rate p in a circuit-level noise model for the $[\![144, 12, 12]\!]$ code. The yellow distributions show the distribution of cluster sizes of the optimal factorization, and the blue distributions are obtained from a BP-guided implementation of an LSD decoder (BP+LSD), where the samples are post-selected on errors for which BP does not converge. That is, errors correctable by BP only are not included here for the BP+LSD distributions. The dashed line represents the average cluster size for the Bethe lattice as in Eq. (6) with the parameter $\theta = 139$.

We expect the LSD cluster distribution to match the cluster distribution on a fault graph with a higher vertex degree than the original fault graph, due to the uncertainty in the growth step guided by the error likelihood vector λ. For the fault graph of the $[\![144, 12, 12]\!]$ code considered here, which has average vertex degree $\theta_\alpha = 103$, and maximum vertex degree $\theta_{\max} = 166$, the fitting parameter $\theta = 139$ supports our heuristic argument. We note that for lower noise rates, specifically when $p < 0.1\%$, the Bethe lattice's average cluster size distribution does not upper bound the LSD distribution. This is consistent with the fact that LSD is never called when the likelihood of error vector λ perfectly describes the actual error, i.e., when BP converges.

In conclusion, for low noise rates, the clusters found by LSD are small and closely approximate the optimal size of the clusters induced by the original error. In the low-error regime, the local decoding problem within each cluster is efficiently solvable.

Supplementary Note 2 – Parallel implementation

In this section, we propose a parallel version of LSD and analyze its time complexity. The central observations are that clusters can be grown and solved (using the on-the-fly elimination technique outlined in Methods 4.2) in parallel and hence potential synchronization issues arise only due to cluster merges.

To enable efficient parallel merging of clusters, we represent the clusters by a parallel version of the union-find data structure as proposed in Ref. [8], where the authors prove that the union-find operations can be performed in parallel polylogarithmic time on each parallel resource with such a data structure. We detail the key steps of the parallel implementation of the LSD algorithm, P-LSD, in Algorithm 1.

1. (Parallel) initialization: we create a cluster C_i for each flipped detector node s_i in the syndrome. We then compute the set of candidate fault nodes at the boundary of each cluster: $\Lambda(C_i) = \Gamma(\{s_i\})$ – see Def 4.3. Clusters are managed as a *union-find* forest in the parallel data structure $\mathbb{U}$, see Ref. [9].

2. (Parallel) pre-growth step: for each invalid cluster in $\mathbb{U}$, the FIND_CANDIDATE function is used to identify, among its candidate fault nodes, the one with the maximum probability to be in error according to the soft information vector λ. We call the set of all these selected nodes growth_faults, one for each invalid cluster.

3. (Parallel + synchronization) growth step: add one new fault node to each cluster and merge colliding clusters [Def. 4.4 (*Cluster collisions*)] using the parallel union-find data structure $\mathbb{U}$ and its efficient UNION-FIND operations. The PARALLEL_UNION routine [8] is used to efficiently determine which set in $\mathbb{U}$ a node belongs to and to efficiently merge any two sets in $\mathbb{U}$ that correspond to colliding clusters.

4. (Parallel) validity check: Apply on-the-fly elimination in parallel for each cluster, see Methods 4.2..

5. Termination: Compute a local solution for each cluster and combine them to return the overall correction.

Algorithm 1: Parallel localized statistics decoding (P-LSD)

H: decoding matrix
$\mathbf{s}$: syndrome support set
λ: fault nodes error soft information
$\mathbb{U}$: parallel union-find data structure
do in parallel for $s_i \in \mathbf{s}$
    $\mathbb{U} \leftarrow$ INITIALIZE_CLUSTER(s_i)
synchronize
while $\exists$ *invalid clusters* **do**
    growth_faults $\leftarrow$ FIND_CANDIDATE($\mathbb{U}$, λ)
    PARALLEL_UNION($\mathbb{U}$, growth_faults)
    do in parallel for invalid $C_i \in \mathbb{U}$
        C_i.ON_THE_FLY_ELIMINATION()

We now analyze the parallel time complexity of the main routine in P-LSD. To this end, we derive a bound on the parallel *depth* of the main routine in P-LSD, where the parallel depth is defined as the maximum number of dependent sequential steps in the computation.

1. Initialization: $O(1)$ parallel depth. This includes the identification of the candidate fault nodes set for each cluster, which has size $O(1)$ for bounded LDPC matrices H.

2. Pre-growth step: iterate over the candidate fault nodes for each cluster, and identify the most suitable fault node given the soft information vector λ. This step can be performed in logarithmic complexity in the size of the boundary using, e.g., a Fibonacci heap. Maintaining a list of candidate fault nodes [Def. 4.3 (*Cluster-boundary*] for each cluster throughout the algorithm incurs only a constant depth overhead.

3. Growth-step: using the methods presented in [8], growing and merging of clusters can be done in $O(\text{polylog}(n))$ depth, where n is the total number nodes in the decoding graph.

4. Validity check: the depth is dominated by the cost of the PLU factorization of each cluster matrix. That is $O(|C_i|^3)$, where $|C_i|$ indicates the number of columns of the cluster $H_{[C_i]}$.

5. Termination: finding a local solution given the PLU factorization of the check matrix of a cluster has cost $O(|C_i|^2)$.

In conclusion, the overall parallel depth for an iteration of the while loop in Line 8 of Algorithm 1 is in $O(\text{polylog}(n) + \kappa^3)$, where κ is the maximum cluster size. Crucially, the maximum cluster size κ is expected to be small for QLDPC codes and low enough error rates, see Section 1. In fact, the average cluster size κ_α can be bounded for regular graphs, such as the Bethe lattice by $\kappa_\alpha \leq \frac{1+p}{1-(\theta-1)p}$, where θ is the vertex degree of the graph and p is the physical noise strength.

The $O(\text{polylog}(n) + \kappa^3)$ depth bound is a loose upper bound and in practice the runtime of the algorithm depends on multiple additional factors such as the concrete implementation, overhead of parallelization, and the number of parallel resources available. The overall *work*, i.e., the total number of steps the algorithm performs across all processes, depends on the number of growth steps. In the worst case, the number of growth steps is proportional to the number of edges in the decoding graph, and the parallel union-find data structure will have overall work almost linear in n, more precisely $O(n\alpha(n))$, where $\alpha(\cdot)$ is the inverse Ackerman function, which is $\alpha(n) \leq 3$ for any practical situation [8, 10, 11]. However, if almost n growth steps were necessary for P-LSD to terminate, the computed error estimate would probably have weight above the percolation threshold of the graph, in which case decoding introduces a non-trivial logical error with high probability. In other words, we expect almost n growth steps only in instances of the decoding problem that have an intrinsically non-local structure, meaning instances that can not be divided into smaller decoding problems. In such a case, there is no benefit in parallelization and hence the parallel runtime complexity is only marginally meaningful.

Supplementary Note 3 – Numerical experiments

In this section, we provide further details on the numerical decoding experiments presented in the main body of the manuscript.

A. Surface code decoding

The surface code [12, 13] is a "matchable" code, that is, qubits participate in at most two X and two Z stabilizer measurements. Hence, syndromes come in pairs and thus the code is decodable using the *minimum-weight perfect matching* (MWPM) algorithm [14, 15]. The surface code is one of the best-known quantum error correcting codes and thus can be seen as a crucial "benchmark" case for decoders. This aspect is facilitated by the high-quality open-source implementation of `Stim` [16]—a tool that can be used for automated circuit noise simulations, which also makes comparing numerical evaluations between different authors consistent. We refer the reader to Refs. [15, 16] for more details on `Stim`.

The BP+UF algorithm [17] is a combination of BP and Union-Find, where the BP soft information is used to guide the cluster growth for the Union-Find algorithm. Note that in the case of a matchable code, the validity check for clusters, which is equivalent to solving systems of linear equations in general, can be replaced by a simple parity computation of the support vector of enclosed, flipped detector nodes and hence is more efficient. Moreover, computing a solution for the Union-Find clusters can be done using a simple algorithm based on spanning-tree construction and thus is also efficient for matchable codes. Note that BP+UF and BP+LSD share an identical cluster growth strategy and therefore should converge to the same set of valid clusters, with the difference that BP+UF applies the peeling decoder [10] for finding a valid correction for each cluster, while the LSD algorithm performs a (partial) inversion of the cluster check matrices $H_{[C]}$ to determine the correction.

B. Hypergraph product codes

We investigate the decoding performance of a family of hypergraph product (HGP) codes [18] with rate $k/n \geq 1/25$. The codes are single-shot decodable [17, 19], that is, a constant-sized decoding window is sufficient for decoding codes of arbitrary size. This family of codes has been previously proposed and investigated in Ref. [20] under a code capacity noise model, and later in Ref. [21] under a circuit-level noise model. In both cases, decoding was achieved by a combination of BP and *small set flip* (SSF) [22]. Additionally, in Ref. [23] the authors investigated this code family under a phenomenological noise model derived from a circuit-level noise model that is inspired by a potential implementation in neutral atom arrays using BP for the bulk of the decoding problem and BP+OSD in the last decoding round to ensure a correction that projects state back into the code space. This family of codes is obtained from random $(3, 4)-$regular Tanner graphs with girth at least 6. Since the construction is random, following Ref. [21], we generate 100 instances of each check matrix and select the best one after performing code capacity simulations. Interestingly, we find that in some cases the performance difference between the best and the worst performing codes is close to a factor of 10. Here, we investigate the sub-threshold performance for a circuit-level noise model for 4 instances of the family using our proposed BP+LSD decoder.

C. Bivariate bicycle codes

We conclude our numerical decoding benchmarks by decoding instances of *bivariate bicycle* (BB) codes recently investigated in Ref. [5], and originally proposed in Ref. [1]. The circuit-level noise simulations use the publicly available implementation by Ref. [24] of the highly optimized syndrome extraction circuits described in Ref. [5]. Detector errors are sampled using `Stim` [16]. Similar to our surface code experiments, we simulate d rounds (where d is the code distance) of syndrome extraction and decode the full syndrome history at once. The BB code family are CSS codes where the X and Z detectors can be decoded separately.

In Figure 6 of the main text, we show the logical Z error rate per syndrome cycle p_{L_Z} for the BB code family decoded with BP+LSD-0. Our results show that BP+LSD-0 achieves comparable decoding performance to the BP+OSD-CS-7 decoding results presented in Ref. [5] (where BP+OSD-CS-7 refers to the "combination sweep" strategy for OSD higher-order processing with order $w = 7$, see Ref. [25]). This implies that, at least for the case of the BB code family, higher-order reprocessing has minimal impact when decoding in the sub-threshold regime. See Section 4 for further discussion of higher-order reprocessing.

Another interesting observation from our numerical decoding simulations of BB codes is that it is sufficient to run BP+LSD with a low number of BP iterations: the BP+LSD simulations shown in Figure 6 of the main text were run with maximum 30 BP iterations, compared to the maximum number of iterations of $10,000$ for the BP+OSD-CS-7 simulations in Ref. [5]. For BP-OSD, it is preferable to run BP until convergence to avoid the cost of computing the inverse of the detector check matrix. In OSD, matrix inversion is performed globally with worst-case cubic runtime complexity in the number of fault nodes in the Tanner graph n. Therefore, the OSD runtime does not scale with the error rate and is independent of the number of flipped detectors. In contrast, this is not the case for our proposed BP+LSD decoder. When run in parallel, the LSD algorithm has an expected runtime in $O(\kappa^3)$, where κ is the maximum cluster size. Further to this, the number of clusters upon initialization is identical to the number of invalid detectors. We therefore expect the decoding time to be proportional to the physical error rate. As such, in the sub-threshold regime, LSD post-processing is much less costly than BP+OSD. As a result, it is not detrimental to the decoder's runtime if a large portion of the computational load is shouldered by the LSD post-processing.

As mentioned in the main text, our implementation of BP+LSD uses BP solely to guide cluster growth. For the codes studied in this work, it is possible to gain sufficient BP soft information after approximately 30 BP iterations.

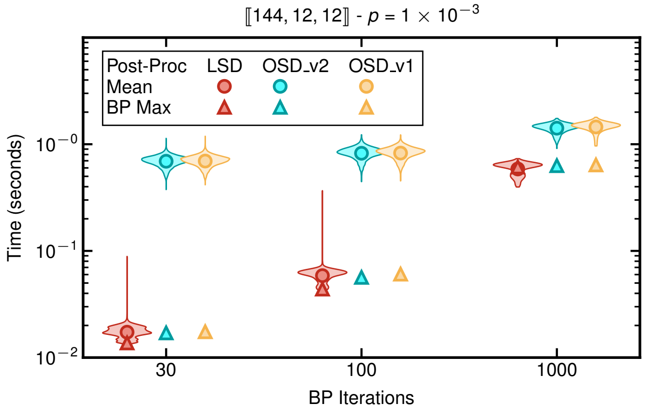

Supplementary Figure 2. Timing statistics for circuit-level noise decoding of the $[\![144, 12, 12]\!]$ bivariate bicycle code at a physical error rate of $p = 0.1\%$. The timing results are post-selected on BP non-convergence. Triangle markers indicate the longest time BP took if it converged.

D. Runtime estimation

In this section, we conduct preliminary simulations to investigate the runtime of the proposed prototypical open-source implementation of the decoder available at [26]. We would like to highlight that we leave an in-depth investigation of a fully parallel and optimized implementation as future work. The results presented here should be seen as a mere representation of the runtime of the currently available software implementation acting as a benchmark for future developments, that is, in particular, it is not meant as a real-time decoder.

Supplementary Figure 2 shows timing statistics for BP+LSD and BP+OSD. The violin plots depict the distributions of the timings of BP+OSD and BP+LSD, where circles represent the means. This data is post-selected on cases where LSD/OSD is called, i.e., where BP does not converge, such that one measures more accurately the performance of the post-processing routines. To highlight the overhead introduced by the post-processor, triangles depict the maximum runtime of BP on shots for which it converged, presenting a lower bound on the achievable timing statistics. For the BP decoder, we have used a parallel schedule and min-sum update rules.

From the plot, it is evident that for the current software implementation of BP+LSD, the LSD step only marginally increases the overall runtime, as opposed to for BP+OSD, where the OSD post-processing time induces significant runtime overhead compared to the runtime of BP alone.

To estimate the runtime behaviour of the decoder in the "realistic scenario", for instance, in a numerical experiment, we also investigate timing data that is not post-selected on the BP-non convergence. Supplementary Figure 3 depicts the total timing statistics of a BP+LSD/OSD call, regardless of BP convergence, i.e., for some samples, BP might converge and LSD/OSD is not called. We observe that with an increased number of BP iterations, the decoding times decrease, perhaps due to more BP convergence and thus fewer post-processor calls. The distributions of BP+OSD exhibit a bimodal form, corresponding to the cases where BP converges or does not. For a larger number of BP iterations, this effect diminished, again perhaps due to the increased convergence rate of BP. This is not the case for BP+LSD, whose distributions do not exhibit this bimodality form because the overhead induced by LSD is (on average) comparable to BP (and lower than OSD), but its distributions feature long tails.

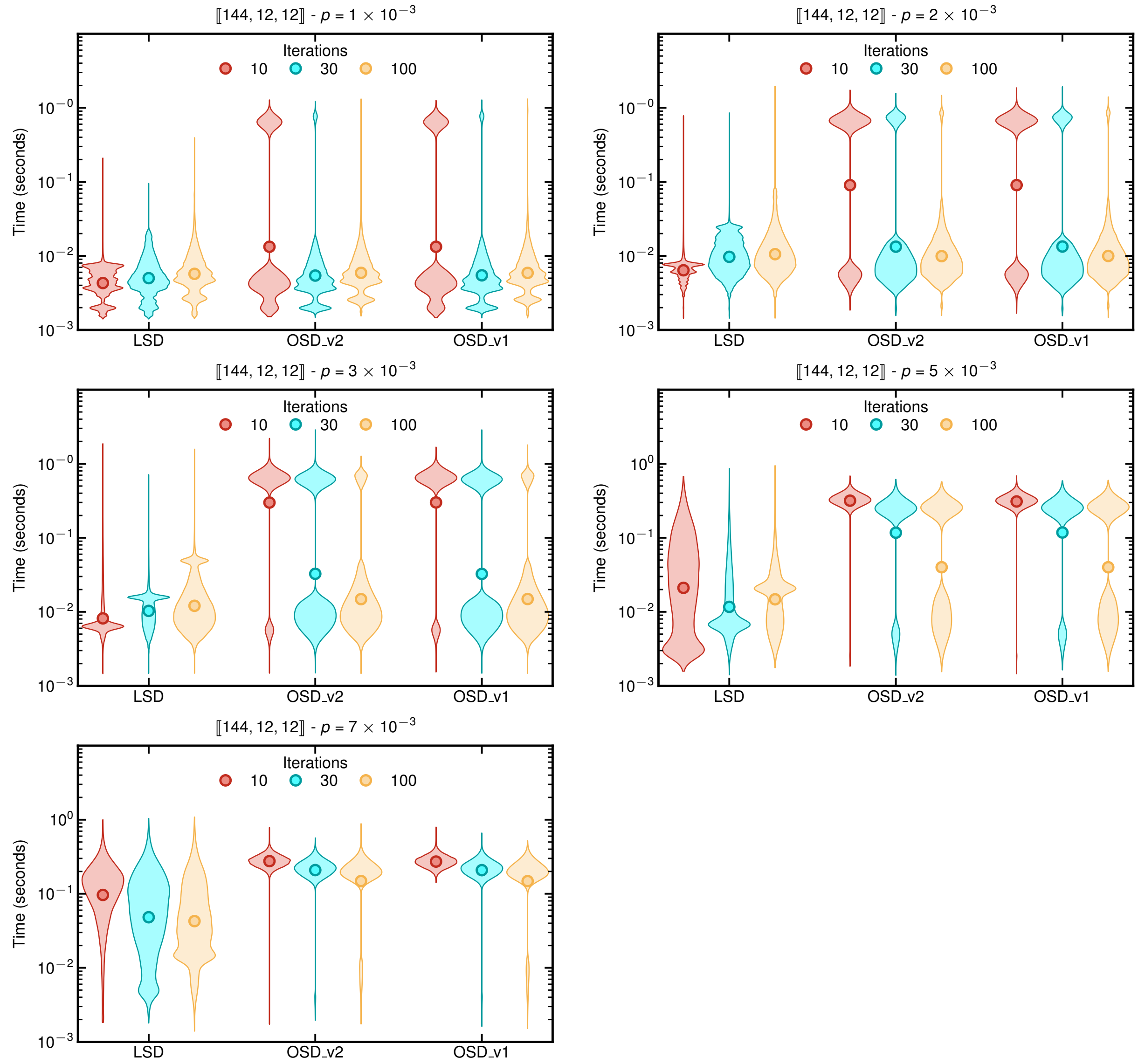

Supplementary Figure 3. Runtime for the decoding of the $[[144, 12, 12]]$ BB code instance at physical noise rate $p = 0.1\%$ (top left), $p = 0.2\%$ (top right), $p = 0.3\%$ (center left), $p = 0.5\%$ (center right), and $p = 0.7\%$ (bottom left), that is, above the pseudo-threshold of the code [5]. Circle markers show average decoding time per shot.

Supplementary Note 4 – Higher-order LSD

Standard LSD achieves comparable decoding performance in terms of logical error rate as BP+OSD. However, improved decoding accuracy can be achieved with higher-order reprocessing, i.e., BP+OSD$-w$ for

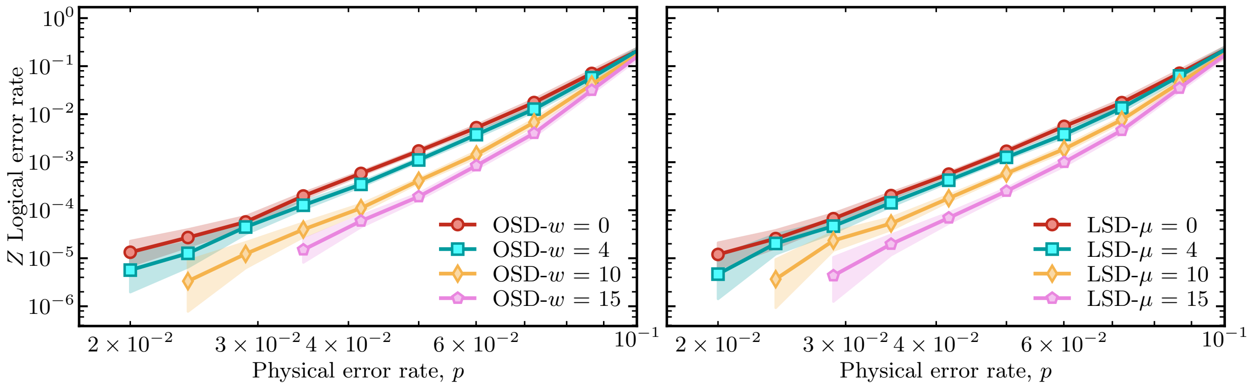

Supplementary Figure 4. Effect of higher-order reprocessing routines when decoding the $[\![882, 48, 16]\!]$ code with (a) BP+OSD$-w$ and (b) BP+LSD$-\mu$. We observe that within the sampling variance, the logical error rates obtained from both decoders are identical.

$w > 0$ [25, 27]. In a similar vein, we propose higher-order LSD, LSD$-\mu$, and denote by LSD-0 standard LSD without further reprocessing.

The central idea of higher-order LSD is to add an additional reprocessing step to LSD-0 to achieve better decoding accuracy. Once LSD-0 has terminated, meaning that all clusters are valid and local solutions have been computed, we conduct, up to μ additional growth step on each valid cluster. On the grown clusters, we then apply standard OSD reprocessing: we do this separately on each cluster matrix and therefore we preserve LSD locality. We note that:

1. The additional growth steps may lead to further cluster merges in general.

2. Clusters cannot become invalid due to the additional growth steps.

3. The number of columns added to each cluster is an important parameter that affects decoding performance.

As such, the choice of the parameter, μ, is important for the runtime as well as the error correction performance of higher-order LSD.

Let us illustrate why it is important for higher-order reprocessing to perform additional growth steps of the clusters once the validity condition $s_{[C_i]} \in \text{IMAGE}(H_{[C_i]})$ is fulfilled. Technically speaking, this is required to ensure that the rank of the union of clusters $H_{[\cup_i C_i]}$ is close to the rank of the full matrix H. Otherwise, not all possible codewords of the classical code H can be represented by the clusters $H_{[C_i]}$, a condition required in higher-order OSD reprocessing. To this end, we apply a heuristic approach where the parameter μ is chosen as a (constant) fraction of the total number of columns of the overall decoding matrix H. If μ is large enough, the cluster matrices have the desired property with high probability. Our numerical findings, which we discuss in the remainder of this section, demonstrate that higher-order LSD can achieve improved decoding performance compared with LSD-0. Moreover, the LSD$-\mu$ performance improvements are on par with performance improvements obtained from global OSD$-w$ reprocessing.

For the local OSD reprocessing in LSD$-\mu$, we use parameters chosen after initial decoding experiments. We leave an in-depth exploration of LSD$-\mu$ and corresponding optimizations open for future work.

a. Comparison of BP+LSD$-\mu$ and BP+LSD$-w$ To demonstrate the effect of LSD$-\mu$, we compare its decoding performance to OSD$-w$ for a lifted product (LP) code instance from Ref. [27], in which the authors

originally proposed BP+OSD$-w$ for decoding QLDPC codes. For demonstration purposes, we perform a code capacity experiment where we sample error vectors $\mathbf{x}$ from an independent and identically distributed noise model, where each qubit is flipped with probability p. Using different decoders, we try to infer whether a logical Z error occurred on any of the logical qubits. For the higher-order reprocessing routine, we use an exhaustive search (OSD-E) of order $w = \mu$ for both LSD$-\mu$ and OSD$-w$. That is, for LSD$-\mu$ we choose, heuristically, the number of additional growth steps per valid cluster to equate the local reprocessing order of the OSD-E routine.

The results depicted in Supplementary Figure 4 demonstrate, on the one hand, that higher-order LSD achieves a lower logical error rate for larger values of μ, and on the other hand, that LSD$-\mu$ can achieve a decoding performance that matches LSD$-w$ within the sampling variance. Note that, however, a direct comparison of the parameters μ and w is not possible since they have different meanings. Additionally, we note that performing 10 or 15 additional growth steps per cluster for the $[\![882, 48, 16]\!]$ code corresponds to growing each cluster additionally by around 1% to 1.5% of the total number of columns in the check matrix H_X, implying that "microdosing" LSD-μ is sufficient.

b. Runtime comparison of higher-order global OSD and higher-order LSD. To estimate the runtime of our serial implementation of BP+LSD$-\mu$ and to compare it against the global BP+OSD$-w$ decoder, we plot the runtime per shot – including the BP iterations and sampling overhead – in seconds for various physical noise rates for the code capacity noise model described in the previous paragraph. The results are shown in Supplementary Figure 5. This figure does not aim to qualitatively highlight the runtime of the decoder, but instead intends to demonstrate that even with the added higher-order reprocessing and additional growth steps, the locality of the decoding problem is, on average, preserved. In fact, if this were not the case, the average decoding time for LSD$-\mu$ would be on par with the decoding time of the global OSD$-w$ decoder. The plot in Supplementary Figure 5 indicates that, even for serial LSD-μ, there is a significant runtime improvement with respect to BP+OSD$-w$, due to the reduced size of the inversion problem, as well as the reduced search space for the OSD-E reprocessing routine on each cluster.

c. Higher-order LSD decoding on bivariate bicycle codes Here, we investigate the decoding performance of LSD$-\mu$ for the three instances of the bivariate bicycle codes from Ref. [5] under the circuit-level noise model described in Methods 4.3. In Supplementary Figure 6 the x-axis represents the additional growth

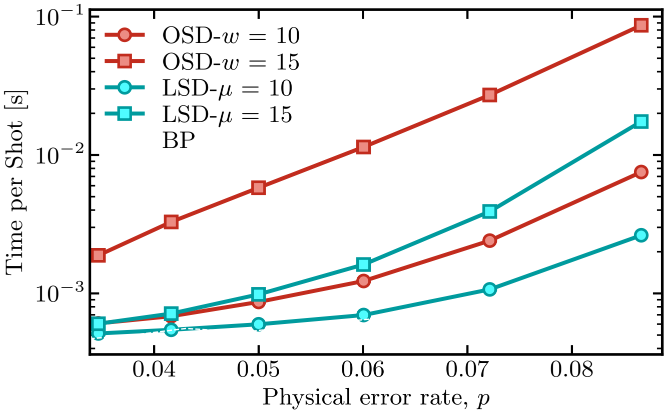

Supplementary Figure 5. The time-per-shot for the $[\![882, 48, 16]\!]$ LP code instance for different orders of OSD and LSD reprocessing. Since our timings include the BP stage of the decoder as well, we find that LSD$-\mu$ with $\mu = 10$ is limited by the serial implementation of the BP decoder and other overhead for low error rates. At larger error rates, its runtime matches the one of BP+LSD-0.

steps per cluster of the LSD$-\mu$ routine as a fraction of the total number of fault nodes in the detector error model. For each data point, we have fixed the local OSD reprocessing method used in LSD$-\mu$ to OSD-CS-4. We observe that for all noise values, the Z logical error rate is consistently reduced by increasing the number of growth steps of LSD$-\mu$. The improvements are relatively small, which is, however, also the case when applying OSD reprocessing globally. Therefore, we explicitly highlight the LSD-0 performance for each code instance and noise strength with horizontal dashed lines. We observe that the largest code, the $[\![144, 12, 12]\!]$ code, benefits the most from the higher-order reprocessing.

d. Adaptive-order reprocessing. It is in principle possible to *adaptively* choose the order of higher-order reprocessing routines based on the observed syndrome $\mathbf{s}$, the soft information vector λ, and the linear dependencies encountered during the construction of the information set I. This is in contrast to current reprocessing routines, that have been implemented in the context of quantum error correction, which fix the order in advance. In the classical error correction literature this adaptive strategy has been described by Fossorier et al. in 1998 [28]. There, the authors describe a (not necessarily efficient) *covering test* that allows, for each received syndrome, to obtain statements about whether an exhaustive search reprocessing routine of order w_2 can improve upon an order $w_1 < w_2$ reprocessing routine. Additionally, assuming that the soft information vector λ closely represents fault probabilities, this test bounds the probability that order w_2 will improve over order w_1 reprocessing. As a result, given a certain target error rate, one can adaptively choose the reprocessing order w to achieve that targeted error rate. We leave the implementation and potential adaption of the covering test to the decoding problem of quantum error correction codes as an interesting open question for future work.

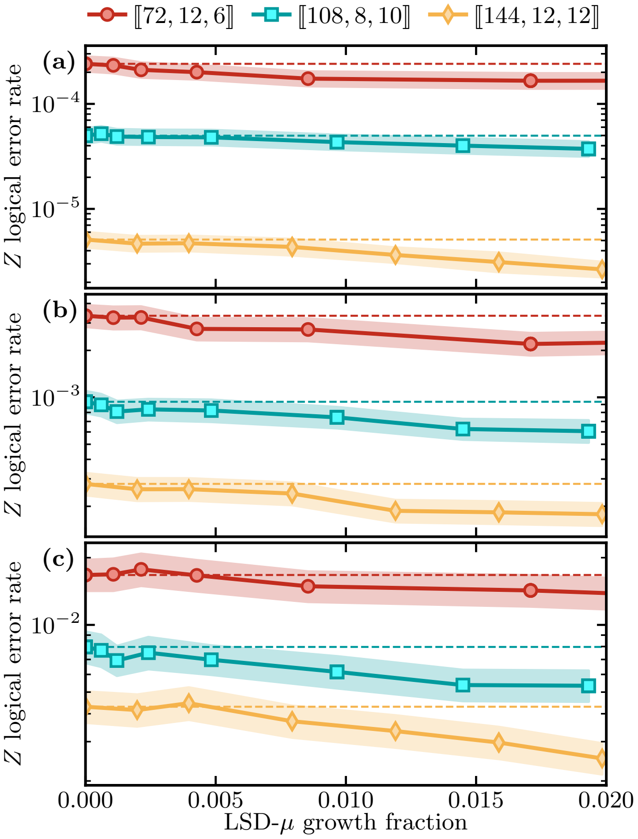

Supplementary Figure 6. Performance improvements of the bivariate bicycle codes of Ref. [5] for higher-order reprocessing with the LSD decoder. Each cluster is grown a certain fraction μ of the total number of fault nodes in the decoding graph after an initial solution to the inversion problem is obtained. Then, a local reprocessing using the standard OSD-CS-4 method is performed. Each panel shows a different error rate of the circuit-level noise model. (a) $p = 0.001$, (b) $p = 0.002$, and (c) $p = 0.003$. The shading indicates hypotheses whose likelihoods are within a factor of 1000 of the maximum likelihood estimate, similar to a confidence interval.

[1] Alexey A. Kovalev and Leonid P. Pryadko. “Quantum Kronecker sum-product low-density parity-check codes with finite rate”. Physical Review A **88**, 012311 (2013).
[2] Nicolas Delfosse and Gilles Zémor. “Upper bounds on the rate of low density stabilizer codes for the quantum erasure channel”. Quantum Information & Computation **13**, 793–826 (2013).
[3] Daniel Gottesman. “Fault-tolerant quantum computation with constant overhead” (2014). arxiv:1310.2984.

[4] Panos Aliferis, Daniel Gottesman, and John Preskill. "Accuracy threshold for postselected quantum computation". Quantum Information & Computation **8**, 181–244 (2008).
[5] Sergey Bravyi, Andrew W. Cross, Jay M. Gambetta, Dmitri Maslov, Patrick Rall, and Theodore J. Yoder. "High-threshold and low-overhead fault-tolerant quantum memory". Nature **627**, 778–782 (2024).
[6] Dietrich Stauffer and Ammon Aharony. "Introduction to percolation theory: Second edition". Taylor & Francis. London (2017). 2 edition.
[7] Geoffrey Grimmett. "Percolation". Springer. Berlin (1989).
[8] Natcha Simsiri, Kanat Tangwongsan, Srikanta Tirthapura, and Kun-Lung Wu. "Work-efficient parallel union-find". Concurrency and Computation: Practice and Experience **30**, e4333 (2018).
[9] Robert Endre Tarjan. "Efficiency of a good but not linear set union algorithm". Journal of the ACM (JACM) **22**, 215–225 (1975).
[10] Nicolas Delfosse and Naomi H. Nickerson. "Almost-linear time decoding algorithm for topological codes". Quantum **5**, 595 (2021).
[11] Robert E. Tarjan and Jan Van Leeuwen. "Worst-case analysis of set union algorithms". Journal of the ACM (JACM) **31**, 245–281 (1984).
[12] Alexei J. Kitaev. "Fault-tolerant quantum computation by anyons". Annals of Physics **303**, 2–30 (2003).
[13] Eric Dennis, Alexei J. Kitaev, Andrew Landahl, and John Preskill. "Topological quantum memory". Journal of Mathematical Physics **43**, 4452–4505 (2002).
[14] Oscar Higgott. "Pymatching: A fast implementation of the minimum-weight perfect matching decoder" (2021). arxiv:2105.13082.
[15] Oscar Higgott and Craig Gidney. "Sparse blossom: correcting a million errors per core second with minimum-weight matching" (2023). arxiv:2303.15933.
[16] Craig Gidney. "Stim: a fast stabilizer circuit simulator". Quantum **5**, 497 (2021). arxiv:2103.02202.
[17] Oscar Higgott and Nikolas P. Breuckmann. "Improved single-shot decoding of higher-dimensional hypergraph-product codes". PRX Quantum **4**, 020332 (2023). arxiv:2206.03122.
[18] Jean-Pierre Tillich and Gilles Zémor. "Quantum LDPC codes with positive rate and minimum distance proportional to the square root of the blocklength". IEEE Transactions on Information Theory **60**, 1193–1202 (2014).
[19] Héctor Bombín. "Single-shot fault-tolerant quantum error correction". Physical Review X **5**, 031043 (2015).
[20] Antoine Grospellier, Lucien Grouès, Anirudh Krishna, and Anthony Leverrier. "Combining hard and soft decoders for hypergraph product codes". Quantum **5**, 432 (2021).
[21] Maxime A. Tremblay, Nicolas Delfosse, and Michael E. Beverland. "Constant-overhead quantum error correction with thin planar connectivity" (2021). arxiv:2109.14609.
[22] Anthony Leverrier and Gilles Zémor. "Quantum Tanner codes" (2022). arxiv:2202.13641.
[23] Qian Xu, J. Pablo Bonilla Ataides, Christopher A. Pattison, Nithin Raveendran, Dolev Bluvstein, Jonathan Wurtz, Bane Vasić, Mikhail D. Lukin, Liang Jiang, and Hengyun Zhou. "Constant-overhead fault-tolerant quantum computation with reconfigurable atom arrays". Nature PhysicsPages 1–7 (2024).
[24] Anqi Gong, Sebastian Cammerer, and Joseph M. Renes. "Toward low-latency iterative decoding of QLDPC codes under circuit-level noise" (2024). arxiv:2403.18901.
[25] Joschka Roffe, David R. White, Simon Burton, and Earl Campbell. "Decoding across the quantum low-density parity-check code landscape". Physical Review Research **2**, 043423 (2020).
[26] "LDPC—tools for building and benchmarking LDPC codes". `https://github.com/quantumgizmos/ldpc` (2024).
[27] Pavel Panteleev and Gleb Kalachev. "Degenerate quantum LDPC codes with good finite length performance". Quantum **5**, 585 (2021).
[28] Marc P. C. Fossorier, Shu Lin, and Jakov Snyders. "Reliability-based syndrome decoding of linear block codes". IEEE Transactions on Information Theory **44**, 388–398 (1998).
